# Improving Cognitive Impairment in Cancer Patients With an Online Course in Mindfulness‐Based Stress Reduction—A Randomized Controlled Trial

**DOI:** 10.1002/pon.70566

**Published:** 2026-08-12

**Authors:** Anna Baumeister, Lea Schuurmans, Lisa Borgmann, Alena Krause, Luca Schmidt, Steffen Moritz, Matthias Rostock

**Affiliations:** ^1^ Department of Psychiatry and Psychotherapy University Medical Center Hamburg‐Eppendorf Hamburg Germany; ^2^ University Cancer Center Hamburg University Medical Center Hamburg‐Eppendorf Hamburg Germany

**Keywords:** cancer‐related cognitive impairment (CRCI), mindfulness‐based stress reduction (MBSR), online course, psycho‐oncology

## Abstract

**Background:**

Cancer patients often suffer from neurocognitive impairments due to cancer treatment, the cancer itself, and/or coping with the illness, significantly impairing quality of life and everyday functioning.

**Aims:**

This study evaluates the effectiveness of an online MBSR course regarding neurocognitive functioning and well‐being of cancer patients.

**Methods:**

A total of 170 cancer patients with cognitive impairments 3 months to 5 years after completing antitumor therapy were randomized to an online MBSR course led by an experienced therapist or a waitlist control group. Assessments were conducted online at baseline (T0) and post‐intervention (T1, 8 weeks). The control group then received the intervention and was assessed again after 8 weeks (T2). Both groups completed a 3‐month follow‐up (T3). The primary outcome was subjective neurocognitive functioning (FACT‐Cog). Secondary outcomes included quality of life (FACT‐G), fatigue (BFI), depressive symptoms (PHQ‐9), attention (Go‐No‐Go), and verbal learning and memory (VLMT). Outcomes were analyzed using mixed models for repeated measures.

**Results:**

Subjective neurocognitive functioning (FACT‐Cog) improved significantly in the intervention group compared to controls at T1 (Cohen's *d* = 0.36–0.72, *p* = 0.004–< 0.001, depending on subscale), as well as quality of life (FACT‐G, Cohen's *d* = 0.32, *p* = 0.001), fatigue (BFI, Cohen's *d* = −0.47, *p* < 0.001), and depressive symptoms (PHQ‐9, Cohen's *d* = −0.57, *p* < 0.001). Objective memory performance (VLMT, Cohen's *d* = 0.25, *p* = 0.030) improved. Effects were maintained at 3‐month follow‐up.

**Conclusions:**

The online MBSR course can effectively improve cancer‐related cognitive impairment and well‐being of cancer patients.

## 1 Introduction

Cancer and its treatments (e.g., chemotherapy, endocrine therapy, immunotherapy) profoundly affect patients' quality of life across physical, psychological, social, and economic domains [1, 2]. Among the wide range of sequelae, cancer‐related cognitive impairment (CRCI), often commonly referred to as “chemobrain” or “chemofog”, represents a particularly compromising consequence reducing daily functioning, work capacity, and overall well‐being [3]. Although originally linked to chemotherapy, CRCI also occurs in patients receiving other types of cancer treatment (e.g., radiation, tyrosine kinase inhibitors, endocrine therapy), suggesting that the underlying mechanisms may relate to the cancer itself rather than to a specific treatment modality [3]. Additionally, psychological coping with the disease might impair cognitive functioning [4]. These impairments often manifest as problems in attention and short‐term memory and can persist long after treatment has ended [3, 5]. A meta‐analysis estimated that approximately one‐third of breast cancer patients experience clinically relevant cognitive difficulties [6].

To mitigate such impairments and improve psychological adjustment, mindfulness‐based interventions (MBIs), particularly Mindfulness‐Based Stress Reduction (MBSR), have gained considerable attention in cancer treatment. Mindfulness is defined as intentional, non‐judgmental awareness of present‐moment experiences [7]. Interventions grounded in this approach have shown efficacy in improving mental and physical health outcomes, including stress reduction, decreased anxiety and depression, and enhanced quality of life among cancer patients [8, 9, 10]. A meta‐analysis including 32 studies reported moderate reductions in anxiety, depression, and fatigue following mindfulness‐based interventions in cancer patients [11]. Similarly, Xie et al. [12] found large effects of MBSR on cancer‐related fatigue, underscoring its potential as a supportive therapy.

Beyond affective benefits, mindfulness may enhance neurocognitive functioning [13, 14], positioning it as a promising intervention for CRCI. Flynn et al. [15] demonstrated that mindfulness‐based programs improve self‐reported neurocognitive functioning in cancer survivors, although their meta‐analysis faced methodological limitations, including small samples, heterogeneous designs across the original studies, and limited inclusion of objective neurocognitive measures. Of the few studies that assessed effects of mindfulness on neurocognitive performance directly, Johns et al. [16] found improved selective attention after mindfulness training in breast and colorectal cancer patients. Duval et al. [17] reported that MBSR reduced subjective (but not objective) memory complaints in breast cancer survivors, whereas Van der Gucht et al. [18] observed enhanced functional brain connectivity in attentional networks and reductions in subjective neurocognitive impairment and fatigue, despite no changes in objective test performance.

Contrasting these encouraging findings, other studies failed to demonstrate superiority of mindfulness interventions over active control conditions or usual care [19, 20]. Melis et al. [20] suggested that simply acknowledging neurocognitive difficulties might alter patients' perception of impairment, thereby influencing self‐reported outcomes even in inactive controls.

Although MBSR is generally well established, its implementation can be challenging for cancer patients due to transportation, fatigue, and treatment‐related physical limitations. Online delivery formats may overcome such barriers, improving accessibility and feasibility [21]. Remote interventions are particularly advantageous for immunocompromised patients, allowing participation from home and minimizing infection risk. Since the onset of the COVID‐19 pandemic, online psychological interventions have become increasingly prevalent and effective [22, 23]. Evidence suggests that digital mindfulness programs can enhance positive affect and reduce stress, anxiety, depression, and sleep problems [24]. However, empirical data from randomized online mindfulness trials in oncology are limited. Chang et al. [22] found that online MBSR improved body image and self‐efficacy in women with breast cancer, whereas Kang et al. [25] reported significant improvements in affective symptoms and sleep. A systematic review by Matis et al. [26] concluded that digital MBIs are generally effective in reducing anxiety, depression, and fatigue among cancer patients. However, oftentimes the interventions used in studies deviate from the original MBSR protocol (by Kabat‐Zinn [27]; i.e., shortened intervention duration, less frequent).

Although mindfulness‐based interventions have shown promise in improving psychological and physical well‐being in cancer survivors, no studies to date have systematically and comprehensively examined the effects of a fully online, standardized, traditional MBSR course on CRCI. To address this gap, the present study evaluated the efficacy of a traditional 8‐week MBSR course delivered entirely online for patients with self‐reported neurocognitive impairments following antitumor treatment. The study's primary outcome was subjective neurocognitive functioning, with secondary outcomes including quality of life, depressive symptoms, fatigue, and objectively assessed neurocognitive performance.

## 2 Methods

The study was conducted as a two‐armed randomized controlled trial with an intervention group (IG) and a waitlist control group (CG). The intervention period was 8 weeks, after which the CG received the intervention, too. For both groups, a follow‐up 3 months after the intervention was conducted. All assessments took place online. Sample size calculation using G*Power indicated an optimal sample size of 192 participants (*α* = 5%, *d* = 0.50, power = 90%), accounting for an estimated 10% dropout rate.

This trial was preregistered in the German Clinical Trials Register (DRKS00027104) and approved by the Local Psychological Ethics Committee (LPEK‐0368).

### 2.1 Procedure and Participants

Outpatients with curatively treated cancer after completion of therapy, as well as stable palliative patients (≥ 6 months), were recruited across Germany via flyers and the University Cancer Center Hamburg website (uke.de/ucch‐mbsr). Inclusion criteria were: (1) neurocognitive impairments resulting from cancer or its treatment; (2) completion of antitumor therapy (chemotherapy, immunotherapy, TKI, antibody, and/or radiotherapy) at least 3 months but not more than 5 years before inclusion; (3) ongoing anti‐hormonal therapy initiated ≥ 3 months prior to inclusion, with no new measures expected within 6 months; (4) age between 18 and 79 years; (5) internet access via PC, laptop, or tablet; (6) sufficient command of the German language; and (7) willingness to participate in an MBSR study. Exclusion criteria were: (1) neurocognitive dysfunction unrelated to malignancy and (2) tumor diseases of the central nervous system (e.g., gliomas, cerebral metastases).

Interested individuals provided consent and completed an initial online screening survey (Qualtrics) assessing demographics, medical history, and neurocognitive functioning. Eligible participants were randomly assigned (1:1) to IG or CG. Self‐reported medical data were validated by medically trained staff via video call. Therapy blocks were scheduled in advance, and randomization occurred prior to each block with up to 16 participants per group. Within two weeks before group start, participants attended a 30‐min introductory interview with the MBSR therapist. The first online assessment (T0) was completed 1 week before intervention start. The IG then began the 8‐week online MBSR course, followed by the post assessment (T1). After T1, the CG received the same course and completed a third survey post‐intervention (T2). For both groups, a 3‐month follow‐up (T3) was conducted after their MBSR course. Enrollment for subsequent blocks ran parallel to the ongoing intervention. The first participant was enrolled on January 7, 2022, and the last completed follow‐up on April 4, 2024, placing recruitment during the COVID‐19 pandemic.

**FIGURE 1:**
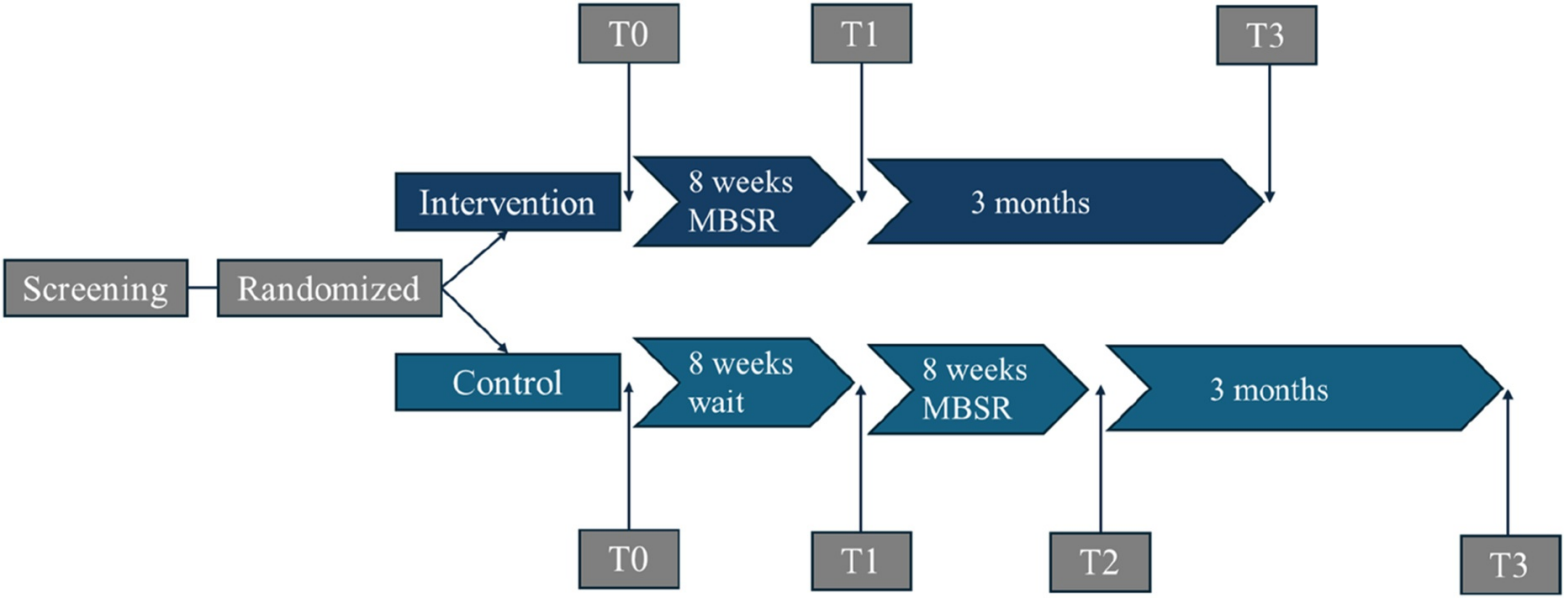
Study procedure per block.

The study procedure per block is shown in Figure 1.

### 2.2 Intervention

The MBSR course conducted in this study was developed by Prof. Dr. Jon Kabat‐Zinn at the University of Massachusetts Medical School (1982). The courses in this study were carried out online and were led by an MBSR teacher with many years of experience who also has extensive experience in supporting people with cancer. In the program, participants learn to apply strategies for stress management and relaxation and to achieve sustainable changes in all areas of life and lifestyle through the principle of mindfulness. The MBSR course covers a period of 8 weeks, with group sessions lasting 2.5 hours once a week and a practice day lasting 7 hours in silence once during the entire period. In addition to practical mindfulness exercises such as body scans, sitting and walking meditations, and mindful movements from yoga, everyday activities such as mindful eating and interpersonal communication are part of the course. Key topics are developed with the group: perception, causes of stress, reactions and behavior, and mindful handling of stress, as well as mindful communication. The course is based on mindful attitudes such as acceptance, patience, non‐judgment, letting go, compassion, and kindness, which are embodied by the course instructor and increasingly integrated by participants as the course progresses. Experiences with meditation practice are explored in inquiry with the group. This exploration of the meditation practice during the course and experiences with practice at home is an essential aspect of the course: Through sharing, course participants realize that they are not alone in their experiences and can directly experience and deepen friendly, compassionate mindfulness with themselves and others in the group. This group dialogue leads to a deepening of the formal and informal practice in daily practice. The formal and informal mindfulness exercises are further deepened in daily practice (approx. 45–60 minutes) with the help of audio instructions and a course manual in order to enable and promote the integration of what has been learned into everyday life.

### 2.3 Measures

#### 2.3.1 Primary Outcome

##### 2.3.1.1 Functional Assessment of Cancer Therapy—Cognitive Function (FACT‐Cog)

The FACT‐Cog [28] is a self‐report instrument assessing perceived neurocognitive function in cancer patients. The 33 items are answered on a Likert‐Scale ranging from 0–4. For each of the four subscales Perceived Cognitive Impairments (CogPCI, 18 items, score range: 0–72), Impact of Perceived Cognitive Impairments on Quality of Life (CogQOL, 4 items, score range 0–16), Comments from Others (CogOTH, 4 items, score range 0–16), and Perceived Cognitive Abilities (CogPCA, 7 items, score range 0–28) a sum score can be obtained. Higher scores indicate better neurocognitive functioning for all subscales, after recoding items of the subscale CogPCA. The FACT‐Cog has been shown to have good internal consistency for all subscales (*α* > 0.80; [29]).

#### 2.3.2 Secondary Outcomes

##### 2.3.2.1 Functional Assessment of Cancer Therapy—General (FACT‐G)

The FACT‐G [30] is a self‐report questionnaire that addresses the four main areas of health‐related quality of life of cancer patients (physical, social, emotional, and functional well‐being) with 27 items in version 4. Items are scored on a 5‐point Likert‐Scale. A total score ranging from 0–108 can be derived by summing up all scores after recoding reversed items so that higher scores indicate better quality of life. The internal consistency of the FACT‐G is 0.89 for the total score [30].

##### 2.3.2.2 Brief Fatigue Inventory (BFI)

The BFI [31] assesses severity of fatigue with nine items on a Likert scale from 0 to 10. A total sum score can be calculated, with higher scores indicating greater fatigue. The BFI demonstrates excellent internal consistency with Cronbach's alpha of 0.96 [32].

##### 2.3.2.3 Patient Health Questionnaire—Depression Module (PHQ‐9)

The PHQ‐9 [33] assesses the severity of depressive symptoms in self‐report with nine items. The items are rated on a 4‐point Likert scale, resulting in a sum score ranging from 0 to 27, with higher scores indicating more severe depressive symptoms. Depression severity is categorized as none or minimal (0–4), mild (5–9), moderate (10–14), and severe (15–27). The PHQ‐9 demonstrates strong internal consistency, with a Cronbach's alpha ranging from 0.86 to 0.89 [33].

##### 2.3.2.4 Go‐No‐Go Test

The Go‐No‐Go test was modeled after the Attention Test Battery [34] for assessing selective attention; the online version has been used before (e.g., [35]). Participants are presented with a series of visual cues. When a previously defined target stimulus (“Go” stimulus, black “x”) appears, the participant is instructed to respond as quickly as possible by a mouse click on the stimulus. Upon appearance of any other stimulus (“No‐Go” stimuli), a response must be withheld. Various stimuli (black “x”, red “x”, black “+”, red “+”) were presented for one second each at the center top of the participant's screen. Following a brief practice session with six stimuli, the main trial commenced, presenting each of the four stimuli 10 times in random order. When a mouse click occurred on a stimulus, it was highlighted with a red square, regardless of the correctness of the response. Errors and misses were recorded but not reported to the participants. The number of hits (correct response to the target stimulus) was used to assess attention.

##### 2.3.2.5 Verbal Learning and Memory Test (VLMT)

Memory performance was modelled after the Verbal Learning and Memory Test (VLMT, [36]), the German version of the Auditory Verbal Learning Test. While the VLMT was originally an auditory test, for the purpose of this online study words were visually presented. The focus was solely on the number of words successfully transferred to long‐term memory (delayed recall), making the presentation method irrelevant. Participants were instructed to memorize 15 words presented at the top center of their screen. Each word was shown individually for 3 seconds. Directly after memorizing, participants were instructed to recall the words in any order by typing each word memorized in a separate text field in the survey. After the first recall, the list was presented again, followed by a recall. At the end of the survey, a final recall after approximately 30 minutes without re‐presenting the list was assessed.

### 2.4 Statistical Analyses

An intention‐to‐treat analysis was conducted using mixed‐effects model for repeated measures (MMRM) for each outcome. In the MMRM, group, time point, and group × time point were considered fixed effects, while also an unstructured covariance matrix for the repeated measures within each participant (ID), allowing for different variances and covariances at each time point, was included. Additionally, the respective baseline values, sex, age, and education were included as covariates in the model. The results of the MMRM are presented as least squares mean differences (LSMD; including the 95% confidence interval [CI]). Additionally, effect sizes were calculated as Cohen's *d*. To evaluate the effectiveness of the intervention, between‐group effects at T1 (T0–T1) are reported. Additionally, within‐group effects after the cross‐over for T1–T2 (post intervention) for the CG and for T0–T3 (3‐month follow‐up) for both groups are reported.

## 3 Results

A total of 223 individuals were screened, of which 52 participants had to be excluded because they either did not meet the inclusion criteria (*n* = 9), were not interested in participation (*n* = 30), or gave no response (*n* = 13). Thus, 171 participants were randomized. The included participants were randomly assigned to either the CG (*n* = 86) or the IG (*n* = 85). Subsequent to randomization, one participant of the IG had to be excluded from the study as he developed brain metastases and therefore was no longer eligible to participate, resulting in a final number of 170 participants (CG *n* = 86, IG *n* = 84) in the analyses. Because of the protracted recruitment phase, the optimal sample size of 192 participants was not reached and recruitment was terminated early.

At post (T1), the retention rate was 95.3% and at follow‐up 3 months after the intervention (T3) 89.5%. In total (both groups) 13 participants (7.6%) did not complete the MBSR course.

**FIGURE 2:**
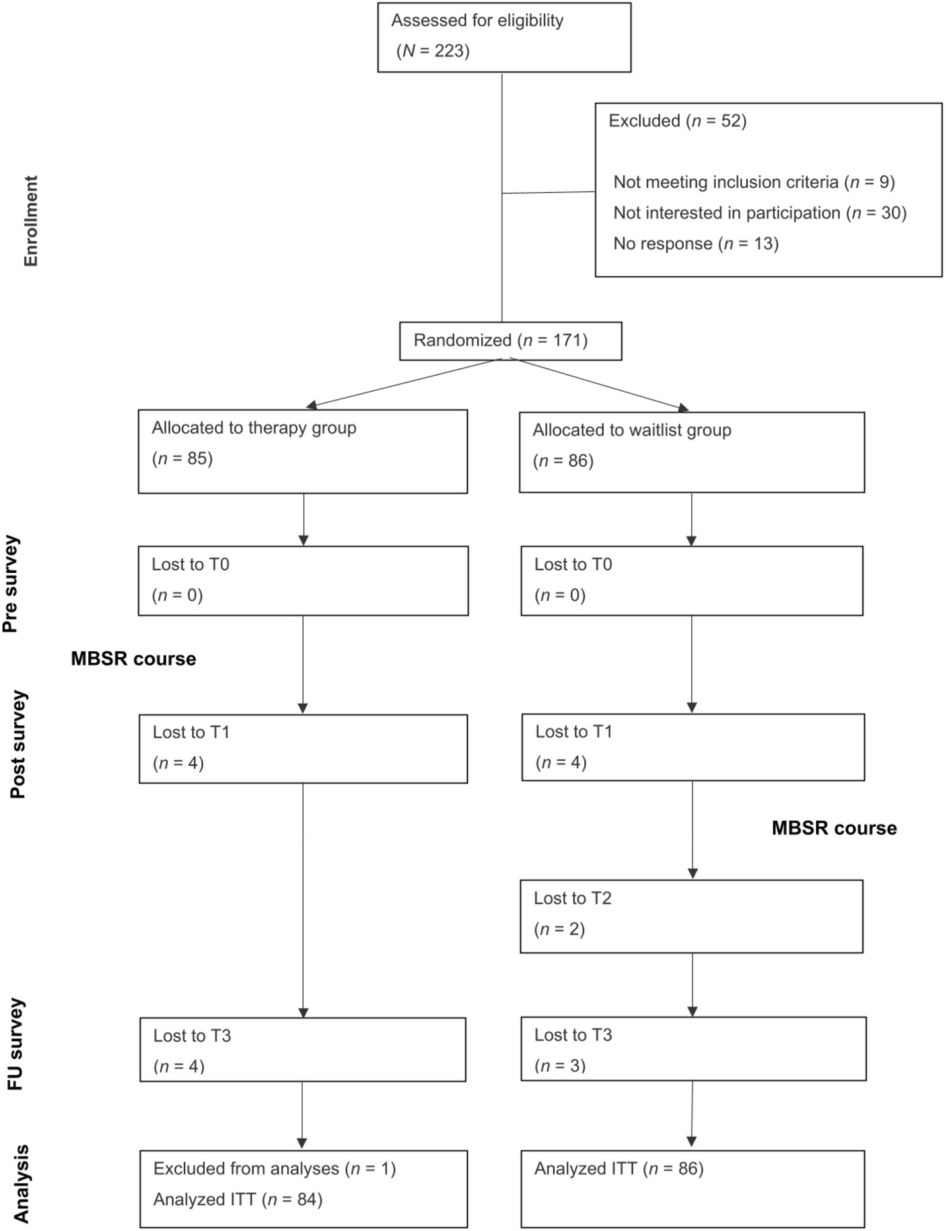
Study flow of the participants.

Participant flow is shown in Figure 2.

The majority of the sample was female (90.6%), with a mean age of 54.4 years (SD = 9.91). Sociodemographic variables and pathological characteristics are shown in Table 1.

**TABLE 1:** Means (SD) and frequencies (%) of sociodemographic and pathological characteristics at baseline.

	Total (*N* = 170)	IG (*n* = 84)	CG (*n* = 86)
Sex (f/m)	154/16 (90.6/9.4%)	78/6 (92.9/7.1%)	76/10 (88.4/11.6%)
Age	54.4 (9.91)	55.2 (9.93)	53.6 (9.90)
Higher education (university degree)	93 (54.7%)	47 (56.0%)	46 (53.5%)
Years since diagnosis	4.4 (3.24)	4.2 (2.87)	4.5 (3.58)
FACT‐Cog			
CogPCI	32.2 (13.06)	31.7 (13.16)	32.7 (13.02)
CogQOL	6.9 (3.51)	7.1 (3.49)	6.7 (3.53)
CogOTH	14.0 (2.11)	14.1 (1.95)	14.0 (2.26)
CogPCA	11.8 (4.49)	12.0 (4.21)	11.6 (4.77)
FACT‐G	62.6 (15.96)	63.1 (15.53)	62.1 (16.44)
Psychopathology			
BFI	52.2 (16.19)	51.6 (15.39)	52.8 (17.01)
PHQ‐9	11.6 (4.50)	11.6 (4.46)	11.6 (4.55)
Objective neurocognitive performance			
Go‐No‐Go	7.9 (3.46)	7.5 (3.63)	8.2 (3.27)
VLMT delay	10.1 (3.08)	10.5 (2.87)	9.8 (3.26)

Abbreviations: BFI = Brief Fatigue Inventory; CG = control group; CogOTH = FACT‐Cog subscale Comments from Others; CogPCA = FACT‐Cog subscale Perceived Cognitive Abilities (recoded); CogPCI = FACT‐Cog subscale Perceived Cognitive Impairments; CogQOL = FACT‐Cog subscale Quality of Life; FACT‐G = Functional Assessment of Cancer Therapy—General; IG = intervention group; PHQ‐9 = Patient Health Questionnaire—9 depression module; VLMT = Verbal Learning and Memory Test delayed recall.

Types of cancer were represented as follows: breast cancer *n* = 104 (61.2%), hematologic disease *n* = 26 (15.3%), ovarian cancer *n* = 13 (7.6%), and others *n* = 27 (15.9%).

### 3.1 Effectiveness

The MMRM showed for the primary between‐group comparison (T0–T1) of the FACT‐Cog subscale scores significantly greater improvements in the IG compared to the CG. This was also found for all secondary endpoints with exception of the Go‐No‐Go task (see Table 2).

**TABLE 2:** Results of the MMRM: Effect sizes (least‐square mean differences and Cohen's *d*) between groups at post‐intervention (T0–T1).

	Between	95% CI		*p*	Cohen's *d*
	LS MD	Lower	Upper		
FACT‐Cog					
CogPCI	9.84	6.77	12.91	< 0.001	0.72
CogQOL	2.38	1.40	3.35	< 0.001	0.60
CogOTH	0.82	0.26	1.37	0.004	0.36
CogPCA	3.50	2.36	4.65	< 0.001	0.71
FACT‐G	5.59	2.42	8.76	0.001	0.32
Psychopathology					
BFI	−8.46	−13.06	−3.86	< 0.001	−0.47
PHQ‐9	−2.87	−3.89	−1.82	< 0.001	−0.57
Objective neurocognitive performance					
Go‐No‐Go	0.48	−0.50	1.47	0.34	0.13
VLMT delay	0.77	0.07	1.46	0.030	0.25

Abbreviations: BFI = Brief Fatigue Inventory; CI = confidence interval; CogOTH = FACT‐Cog subscale Comments from Others; CogPCA = FACT‐Cog subscale Perceived Cognitive Abilities (recoded); CogPCI = FACT‐Cog subscale Perceived Cognitive Impairments; CogQOL = FACT‐Cog subscale Quality of Life; FACT‐G = Functional Assessment of Cancer Therapy—General; LS MD = least square mean difference; PHQ‐9 = Patient Health Questionnaire—9 depression module; VLMT = Verbal Learning and Memory Test delayed recall.

**FIGURE 3:**
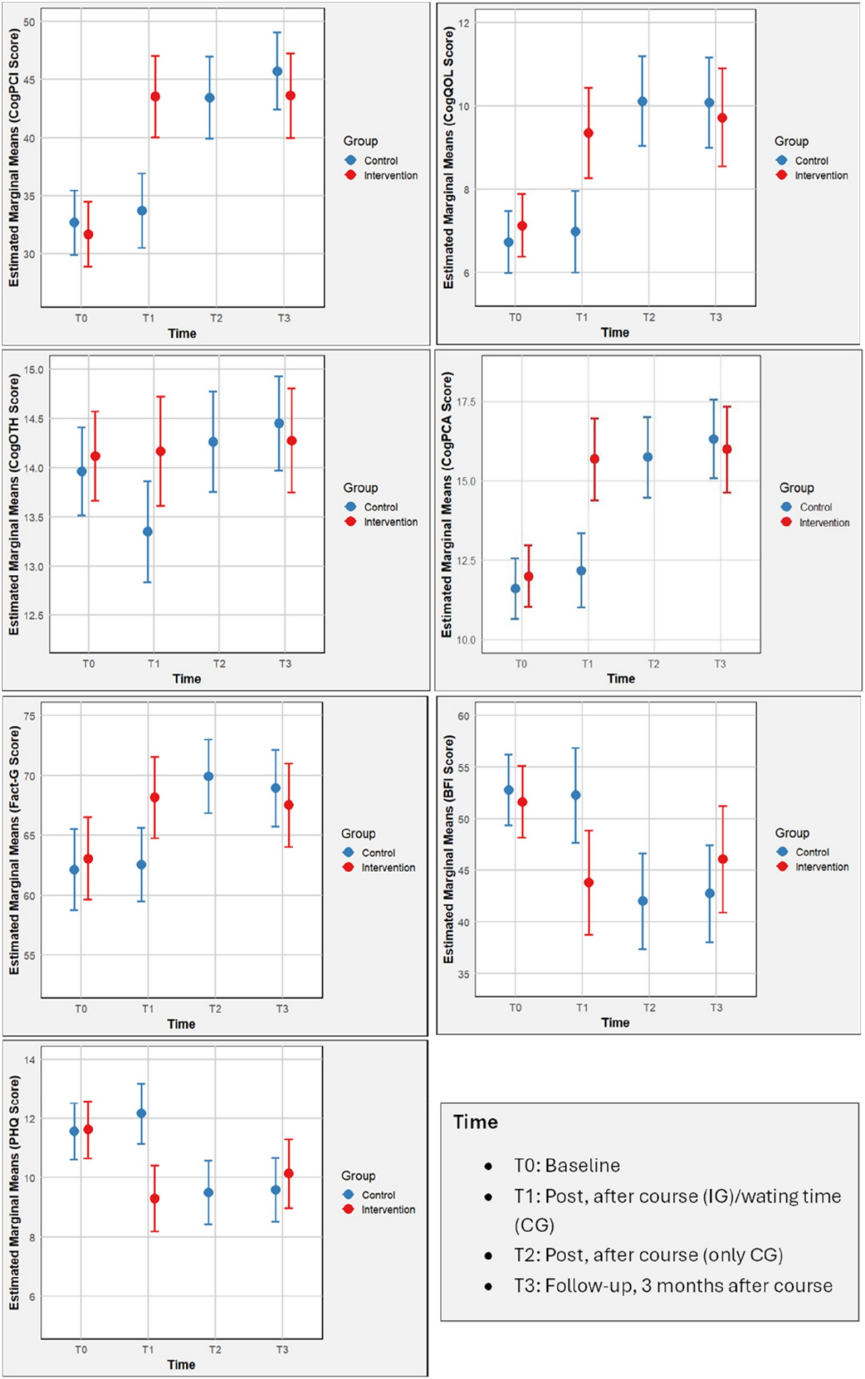
Estimated marginal means of the outcomes at all assessments in both groups over the study period (only CG was assessed at T2).

The treatment effect was supported by within‐group effects of the CG between T1 and T2, showing significant improvement in all endpoints after the intervention, again with the exception of the Go‐No‐Go task (see Table 3). Estimated marginal means of the outcomes at all assessments in both groups are shown in Figure 3. Within‐group effects for both groups at T3 showed significant changes compared to T0, indicating that treatment effects were sustained over the 3‐month follow‐up period (all *p* < 0.05; see Supporting Information S1: Table S1 and Table S2), with the exception of the Go‐No‐Go Task in the CG (CG: *p* = 0.989). Go‐No‐Go task in the IG, however, did improve significantly at follow‐up (*p* = 0.015).

**TABLE 3:** Results of the MMRM: Within‐group effect sizes (least‐square mean differences and Cohen's *d*) for the waitlist control group post‐intervention (T1–T2).

	Within T1–T2 CG	95% CI		*p*	Cohen's *d*
	LS MD	Lower	Upper		
FACT‐Cog					
CogPCI	9.76	7.10	12.42	< 0.001	0.75
CogQOL	3.16	2.09	4.22	< 0.001	0.89
CogOTH	0.92	0.32	1.51	0.001	0.41
CogPCA	3.57	2.54	4.59	< 0.001	0.75
FACT‐G	7.36	4.37	10.36	< 0.001	0.45
Psychopathology					
BFI	−10.32	−14.21	−6.43	< 0.001	−0.61
PHQ‐9	−2.70	−3.85	−1.54	< 0.001	−0.59
Objective neurocognitive performance					
Go‐No‐Go	0.73	−0.14	1.60	0.13	0.22
VLMT delay	1.63	0.99	2.27	< 0.001	0.50

Abbreviations: BFI = Brief Fatigue Inventory; CG = Waitlist control group; CI = confidence interval; CogOTH = FACT‐Cog subscale Comments from Others; CogPCA = FACT‐Cog subscale Perceived Cognitive Abilities (recoded); CogPCI = FACT‐Cog subscale Perceived Cognitive Impairments; CogQOL = FACT‐Cog subscale Quality of Life; FACT‐G = Functional Assessment of Cancer Therapy—General; LS MD = least square mean difference; PHQ‐9 = Patient Health Questionnaire—9 depression module; VLMT = Verbal Learning and Memory Test delayed recall.

## 4 Discussion

Neurocognitive impairments are a common challenge among cancer patients, and mindfulness‐based interventions have shown promise in improving neurocognitive and psychological functioning. This randomized controlled trial evaluated the effectiveness of a traditional 8‐week fully online MBSR course on neurocognitive impairments with a large sample of patients with treated cancer.

The intervention led to significant improvements in subjective CRCI (FACT‐Cog, primary endpoint) compared to the waitlist control group. Additionally, participants reported better quality of life, reduced depressive symptoms, and less fatigue post‐intervention, with effect sizes ranging from *d* = 0.32–0.72. After receiving the intervention, the control group, too, showed comparable within‐subject improvements, all of which remained stable over a 3‐month follow‐up. Improvements on the FACT‐Cog subscale CogOTH, reflecting others' perceptions of patients' cognition, were statistically significant but small, possibly due to limited social contact during the COVID‐19 pandemic, which reduced opportunities for external evaluation of neurocognitive performance.

These findings align with previous studies reporting benefits of MBI and MBSR for cancer patients [11, 12, 15] and extend this evidence by showing that a fully online MBSR format can yield outcomes comparable to traditional in‐person programs. The online format offers clear advantages in accessibility, reducing barriers related to travel and physical attendance while maintaining the intensity of the standard 8‐week program.

Contrasting results have been reported in other trials. For example, Melis et al. [20] found no significant effects of mindfulness training compared to active and waitlist controls, possibly due to the shorter disease duration of their participants (2 vs. 4.4 years in our sample), which may have allowed for more spontaneous improvement. In our study, improvements in the control group occurred only after completing the intervention, not during the waiting period, supporting the specific benefit of MBSR. Similarly, Lengacher et al. [19] found no significant effects, though their 6‐week adaptation of MBSR may have reduced treatment intensity. In contrast, Duval et al. [17] and Van der Gucht et al. [18] reported positive effects of MBSR on CRCI, but both investigated small samples of female breast cancer survivors and applied modified or non‐traditional MBSR protocols. The present study, using a standardized 8‐week course delivered entirely online, thus contributes novel evidence supporting both the feasibility and effectiveness of this approach.

The effects observed across the subjective outcome parameters in this study, particularly in comparison to previous research, may be attributable to several factors. First, the full MBSR program in its original form was implemented, which is likely more effective than the modified or abbreviated versions commonly used in prior studies. Other existing trials rely on adapted protocols that may not fully capture the therapeutic potential of MBSR. Second, the high level of expertise of the MBSR therapist in this study might have played a critical role. The therapist not only had extensive experience in teaching meditation practices but also substantial clinical experience working with cancer patients. This combination of methodological fidelity and therapist expertise may have enhanced both participant engagement and intervention efficacy, thereby contributing to the effects observed. Effects on objectively assessed cognition were less consistent. Memory performance (VLMT) improved significantly in the intervention group compared to controls, but only with a small effect size (*d* = 0.25). As the same word list was used in each session, improvements may reflect practice effects rather than true neurocognitive change. Selective attention, assessed with the Go‐No‐Go test, did not improve significantly. These findings should be interpreted cautiously, as all neuropsychological assessments were conducted remotely without standardized supervision, potentially compromising validity.

The discrepancy between subjective and objective neurocognitive outcomes mirrors previous findings in CRCI and affective disorders [6, 37]. Subjective impairments often reflect emotional distress more than objective deficits [6, 38]. Affective variables, such as depression, predict subjective neurocognitive complaints more strongly than neuropsychological test performance [39]. Thus, improvements in perceived neurocognition in this study may partly reflect reduced depressive symptoms. Future research should explore whether mood improvements mediate cognitive outcomes in mindfulness‐based interventions.

### 4.1 Implications

The present findings suggest that fully online MBSR may be a feasible and clinically useful intervention for cancer patients experiencing subjective cancer‐related cognitive impairment. Given its positive effects on perceived cognitive functioning, quality of life, depressive symptoms, and fatigue, online MBSR could be considered as a supportive care option, particularly for patients who face barriers to attending in‐person programs due to physical limitations, transportation, or limited availability of local services. Clinicians should, however, be mindful that improvements in subjective cognition may partly reflect reductions in emotional distress rather than changes in objective cognitive performance. Therefore, online MBSR may be especially valuable as part of a broader psychosocial care approach targeting both cognitive complaints and psychological well‐being in cancer patients.

### 4.2 Limitations

Some limitations of this study must be mentioned. Firstly, the sample consisted predominantly of women with breast cancer. Although other types of cancer were also represented, generalizability of the effects is therefore limited. Furthermore, only the effects at T1 allow a randomized controlled comparison between the intervention and control groups, as the control group received the intervention after T1. Since this study did not include an active control condition, it cannot be ruled out that the observed effects are non‐specific to the intervention and may, for example, be attributable to general factors such as group contact or social support. In addition, all courses were conducted by the same therapist. Thus, it cannot be excluded that a therapist‐specific effect occurred, meaning that the observed effects might differ if the program were delivered by other instructors. Another limitation concerns the assessment of objective cognitive measures, which were conducted online via self‐report and therefore did not correspond to a standardized, supervised testing environment. Completing neuropsychological tests at home in front of one's own computer may have reduced the validity and reliability of these results.

## 5 Conclusion

This randomized controlled trial demonstrates that an 8‐week fully online MBSR course significantly improved subjective cancer‐related cognitive impairment, as well as quality of life, depressive symptoms, and fatigue in a large sample of cancer patients. The effects remained stable over 3 months, indicating lasting benefits. These findings suggest that a traditional MBSR course, even when delivered entirely online, can effectively enhance perceived neurocognitive functioning and psychological well‐being while improving accessibility. However, results for objectively measured cognition were less consistent and may reflect limitations in the unsupervised online testing format.

Overall, online MBSR appears to be a feasible and beneficial intervention for cancer survivors with neurocognitive complaints, enhancing various aspects of neurocognitive and emotional well‐being in cancer patients, thereby potentially improving their overall quality of life and everyday functioning.

## Author Contributions

**Anna Baumeister:** methods, formal analysis, writing – original draft. **Lea Schuurmans:** formal analysis, writing – review and editing. **Lisa Borgmann:** writing – original draft. **Alena Krause:** project administration, writing – review and editing. **Luca Schmidt:** project administration, writing – review and editing. **Steffen Moritz:** methods, supervision, writing – review and editing. **Matthias Rostock:** project administration, methods, writing – review and editing, supervision.

## Funding

This study was supported by Stifterverband für die Deutsche Wissenschaft, Baedeker Strasse 1, 45128 Essen, Germany and Hamburger Krebsgesellschaft e.V., Butenfeld 18, 22529 Hamburg, Germany.

## Conflicts of Interest

The authors declare no conflicts of interest.

## Supporting information


Supporting Information S1

## Data Availability

The data that support the findings of this study are available on request from the corresponding author. The data are not publicly available due to privacy or ethical restrictions.
