# Supplementary material for: Improving Cognitive Impairment in Cancer Patients With an Online Course in Mindfulness‐Based Stress Reduction—A Randomized Controlled Trial
Source: Psychooncology. 2026 Aug 12;35(8):e70566. doi: 10.1002/pon.70566 (PMC13463220; doi:10.1002/pon.70566)
Supplement: Supplementary file 1 [file PON-35-e70566-s001.docx]

**SUPPLEMENTS**

**Table S1**. Results of the MMRM: Within-group effect sizes (least-square mean differences and Cohens’s *d*) for the waitlist control group at follow-up (3 months after receiving the intervention; T0-T3).

|  | **Within CG**  **T0 – T3** | **95% CI** | |  |  |
| --- | --- | --- | --- | --- | --- |
|  | **LS MD** | **Lower** | **Upper** | ***p*** | **Cohen’s *d*** |
| ***FACT-Cog*** |  |  |  |  |  |
| **CogPCI** | 13.72 | 10.78 | 16.67 | <.001 | 1.05 |
| **CogQOL** | 3.48 | 2.30 | 4.67 | <.001 | 0.99 |
| **CogOTH** | 0.83 | 0.21 | 1.46 | 0.004 | 0.37 |
| **CogPCA** | 5.56 | 3.45 | 5.67 | <.001 | 0.96 |
| **FACT-G** | 7.26 | 4.05 | 10.46 | <.001 | 0.44 |
| ***Psychopathology*** |  |  |  |  |  |
| **BFI** | –9.26 | –13.98 | –4.54 | <.001 | –0.54 |
| **PHQ-9** | –2.73 | –3.89 | –1.57 | <.001 | –0.60 |
| ***Objective neurocognitive***  ***performance*** |  |  |  |  |  |
| **Go-No-Go** | 0.14 | –1.01 | 1.29 | 0.989 | 0.04 |
| **VLMT Delay** | 2.54 | 1.92 | 3.16 | <.001 | 0.78 |

*Note*. CG = Waitlist control group; LS MD = least square mean difference; CI = confidence interval; CogPCI = FACT-Cog subscale Perceived Cognitive Impairments; CogQOL = FACT-Cog subscale Quality of Life; CogOTH = FACT-Cog subscale Comments from Others; CogPCA = FACT-Cog subscale Perceived Cognitive Abilities (recoded); FACT-G = Functional Assessment of Cancer Therapy – General; BFI = Brief Fatigue Inventory; PHQ-9 = Patient Health Questionnaire – 9 depression module; VLMT = Verbal Learning and Memory Test delayed recall.

**Table S2**. Results of the MMRM: Within-group effect sizes (least-square mean differences and Cohens’s *d*) for the intervention group at follow-up (T0-T3).

|  | **Within IG**  **T0 – T3** | **95% CI** | |  |  | |  |
| --- | --- | --- | --- | --- | --- | --- | --- |
|  | **LS MD** | **Lower** | **Upper** | ***p*** | | **Cohen’s *d*** | |
| ***FACT-Cog*** |  |  |  |  | |  | |
| **CogPCI** | 11.73 | 8.29 | 15.17 | <.001 | | 0.89 | |
| **CogQOL** | 2.89 | 1.86 | 3.93 | <.001 | | 0.83 | |
| **CogOTH** | 0.64 | 0.18 | 1.09 | 0.004 | | 0.33 | |
| **CogPCA** | 4.00 | 2.75 | 5.24 | <.001 | | 0.95 | |
| **FACT-G** | 5.83 | 3.03 | 8.63 | <.001 | | 0.38 | |
| ***Psychopathology*** |  |  |  |  | |  | |
| **BFI** | –5.20 | –9.41 | –0.99 | .011 | | –0.34 | |
| **PHQ-9** | –2.24 | –3.24 | –1.24 | <.001 | | –0.50 | |
| ***Objective neurocognitive***  ***performance*** |  |  |  |  | |  | |
| **Go-No-Go** | 1.06 | 0.17 | 1.95 | .015 | | 0.29 | |
| **VLMT Delay** | 2.21 | 1.56 | 2.86 | <.001 | | 0.77 | |

*Note*. IG = intervention group; LS MD = least square mean difference; CI = confidence interval; CogPCI = FACT-Cog subscale Perceived Cognitive Impairments; CogQOL = FACT-Cog subscale Quality of Life; CogOTH = FACT-Cog subscale Comments from Others; CogPCA = FACT-Cog subscale Perceived Cognitive Abilities (recoded); FACT-G = Functional Assessment of Cancer Therapy – General; BFI = Brief Fatigue Inventory; PHQ-9 = Patient Health Questionnaire – 9 depression module; VLMT = Verbal Learning and Memory Test delayed recall.
